# Life as a Vector of Dengue Virus: The Antioxidant Strategy of Mosquito Cells to Survive Viral Infection

**DOI:** 10.3390/antiox10030395

**Published:** 2021-03-05

**Authors:** Chih-Chieh Cheng, Eny Sofiyatun, Wei-June Chen, Lian-Chen Wang

**Affiliations:** 1 Graduate Institute of Biomedical Sciences, Chang Gung University, Kwei-San, Tao-Yuan 33332, Taiwan; jengjyhjay@gmail.com (C.-C.C.); enysofi@gmail.com (E.S.); 2 Program in Tropical Medical Science, Gadjah Mada University, Yogyakartan 53482, Indonesia; 3 Department of Public Health and Parasitology, Chang Gung University, Kwei-San, Tao-Yuan 33332, Taiwan; 4 Molecular Infectious Disease Research Center, Chang Gung Memorial Hospital, Kwei-San, Tao-Yuan 33305, Taiwan

**Keywords:** dengue virus, mosquito cells, antioxidant defense, survival strategy

## Abstract

Dengue fever is a mosquito-borne viral disease of increasing global importance. The disease has caused heavy burdens due to frequent outbreaks in tropical and subtropical areas of the world. The dengue virus (DENV) is generally transmitted between human hosts via the bite of a mosquito vector, primarily *Aedes aegypti* and *Ae. albopictus* as a minor species. It is known that the virus needs to alternately infect mosquito and human cells. DENV-induced cell death is relevant to the pathogenesis in humans as infected cells undergo apoptosis. In contrast, mosquito cells mostly survive the infection; this allows infected mosquitoes to remain healthy enough to serve as an efficient vector in nature. Overexpression of antioxidant genes such as superoxide dismutase (SOD), catalase (CAT), glutathione peroxidase (GPx), glutathione S-transferase (GST), glutaredoxin (Grx), thioredoxin (Trx), and protein disulfide isomerase (PDI) have been detected in DENV2-infected mosquito cells. Additional antioxidants, including GST, eukaryotic translation initiation factor 5A (eIF5a), and p53 isoform 2 (p53-2), and perhaps some others, are also involved in creating an intracellular environment suitable for cell replication and viral infection. Antiapoptotic effects involving inhibitor of apoptosis (IAP) upregulation and subsequent elevation of caspase-9 and caspase-3 activities also play crucial roles in the ability of mosquito cells to survive DENV infection. This article focused on the effects of intracellular responses in mosquito cells to infection primarily by DENVs. It may provide more information to better understand virus/cell interactions that can possibly elucidate the evolutionary pathway that led to the mosquito becoming a vector.

## 1. Introduction

Dengue viruses (DENVs) are members of the genus *Flavivirus* belonging to the family Flaviviridae [1]; these viruses are naturally transmitted between humans via the bite of a mosquito vector. The virion is a spherical particle with a size of 30–50 nm in diameter; its genome contains a positive-sense single-strained RNA consisting of ~10,700 nucleotides which is packed inside an icosahedral nucleocapsid that is covered by an envelope protein [2]. The gene products consist of 3 structural proteins (capsid; C, membrane; M/PrM, and envelope; E), and 7 non-structural proteins (NS1, NS2A, NS2B, NS3, NS4A, NS4B, and NS5) are produced via cleavage of a polyprotein [3]. Further, DENVs can be classified into 4 serotypes (DENV1–4) based on antigenic differences; each serotype causes dengue fever with indistinguishable febrile symptoms [4]. In addition to dengue fever, dengue infection sometimes causes severe illness, including dengue hemorrhagic fever (DHF) and dengue shock syndrome (DSS) [5]. Dengue-associated encephalitis has occasionally been reported in some cases [6]. As there is a large proportion of subclinical infections, the infection may sometimes be overlooked, and such silent transmission facilitates the occurrence of outbreaks [7,8].

Dengue fever has increased in global importance due to heavy economic burdens from frequent epidemics in most tropical and subtropical regions of the world [9,10]. It is estimated that at least 2.5 billion people in about 129 countries are at risk of dengue infections; among these, an estimated 50 million cases annually occur globally [11]. Thus far, specific antiviral therapies have not been demonstrated to be efficacious. Meanwhile, the absolute safety of an effective vaccine for dengue prevention remains to be clarified, since the first approved dengue vaccine, Dengvaxia^®®^ (CYD-TDV), was licensed in December 2015 [12]. Currently, prevention of dengue transmission in most endemic or epidemic areas depends on the effective control of mosquito vectors [13].

Like other mosquito-borne viral diseases, a network constructed by the mosquito, virus, and human or other vertebrate hosts has formed through the long-term process of coevolution [14]. The natural life cycle of DENVs is believed to have its origin in the transmission cycle involving *Aedes* mosquitoes and monkeys in jungle environments [15]. The population density and spatial-temporal distribution of mosquito vectors are highly dependent on climatic factors that include temperature, precipitation, and humidity [16,17]. In most areas with dengue outbreaks in the world, *Aedes aegypti* and/or *Ae. albopictus* are reported to be principal vectors, because both species are susceptible to viral infection and frequently choose humans as targets for a blood meal [18,19]. Clearly, the mosquito vector must provide a suitable place for viral replication. In addition, prolonged survivability of the mosquito vector is obviously essential to produce large amounts of progeny virions [20]. It is now known that the fate of infected mosquito cells can be rectified via a wide spectrum of fundamental cellular processes, particularly those related to mosquito defense mechanisms which are able to cope with stresses induced by an infection [21,22,23]. In fact, gene cross-talk was recently identified in the *Ae. aegypti* mosquito with DENV infection, and a role required for its successful defense against infection was revealed [24]. In particular, mosquito cells in midgut tissues mostly remain intact after a period of infection by the virus [25]. Understanding the mechanism in terms of the survival of the mosquito from DENV infection may offer an avenue to explore how the mosquito originally became a disease vector. Herein, we describe at the molecular level how the mosquito can tolerate DENV infection, which is required for sustainability of the natural cycle of viral replication and transmission.

## 2. Mosquitoes Which Are Able to Transmit DENV

It is known that *Ae. aegypti* and *Ae. albopictus* are principal vectors of DENVs. Effective mosquito vector control is beneficial in reducing dengue outbreaks; therefore, failure to control their populations may result in disease resurgence [26]. More specifically, dengue outbreaks mostly occur in regions where *Ae. aegypti* is abundant or the only species distributed [27,28,29]. This indicates that *Ae. aegypti* plays a major role in transmitting DENVs, as well as yellow fever, Zika, and chikungunya. Therefore, there is an urgent need to target this important population to efficiently control it and the diseases it transmits.

*Aedes aegypti* originated in Africa, but is now prevalent in most tropical and subtropical areas of the world [30]. Geographically, it is distributed in Africa, Asia, the Pacific, Central and South America, and a portion of North America [31,32,33]. As with most mosquitoes, its population size and seasonal fluctuation are highly influenced by climate, including temperature, rainfall, and relative humidity [34,35]. *Aedes albopictus*, also known as the “Asian tiger mosquito”, is a species that can serve as an alternate dengue vector. Several ecological characteristics have been discriminated between *Ae. aegypti* and *Ae. albopictus*. Of these, an exophilic breeding preference, exophagic biting activity, opportunistic or non-anthropophilic feeding behavior, and less-frequent blood-feeding in a single gonotrophic cycle are characteristics possessed by *Ae. albopictus* [36,37]. These cause *Ae. albopictus* to be less competent in transmitting DENVs, and it is thus a secondary or minor vector of dengue fever [38,39]. Despite *Ae. albopictus* being classified as a secondary dengue vector, it was involved in dengue outbreaks that occurred in southern China several years ago [40]. From the viewpoint of global health, its role as a dengue vector should not be neglected. As a matter of fact, several small-scale dengue outbreaks were also reported from regions in Taiwan where only *Ae. albopictus* breeds [41]. *Aedes albopictus* was originally prevalent in Southeast Asia and Pacific islands [42]. Nevertheless, it has become extensively distributed in most parts of Asia, North and South America, Europe, and Africa since its first appearance in Texas, USA as early as the 1980s [43,44].

*Aedes albopictus* was found to breed in areas with lower temperatures during the winter, compared to *Ae. aegypti*. Investigations of the geographical distributions revealed that its distribution is more prevalent in areas with cooler temperatures. Cold tolerance is a critical biological parameter that determines the distribution of mosquitoes [45]. As with many other insects, diapause is known to be an alternative life-history strategy that helps some species of mosquito successfully overwinter [46]. Diapause is a dominant feature in the life history of many species of mosquito. It provides a mechanism for bridging seasons that are unfavorable for the mosquito to live and survive; disease transmission cycles may thus be affected [47]. As a matter of fact, distinct embryogenic kinetics were revealed in tropical and temperate strains of *Ae. albopictus.* Eventually, increased survival of eggs was seen in a temperate strain of *Ae. albopictus* during the winter [48,49]. It seems that embryonic diapause is a striking factor resulting in the rapid global expansion of *Ae. albopictus* [50]. Lacking embryonic diapause may have restricted *Ae. aegypti* to only breed in the southern part of Taiwan, i.e., below the Tropic of Cancer [41]. On the other hand, cold tolerance during the winter allows *Ae. albopictus* to be distributed island-wide. This unique phenomenon could account for the unequal distribution of dengue outbreaks; in past decades, relatively few dengue outbreaks were reported from Northern Taiwan where *Ae. albopictus* breeds, and it is the only species considered to be a potential dengue vector in that region [51].

## 3. Infection, Dissemination, and Effects of the DENV in the Mosquito

Mosquito and human are alternate natural hosts involved in the transmission cycle of DENVs and many other arboviruses. Viral proliferation in the mosquito vector begins with a blood meal from a patient in the acute stage of infection. The ingested virus can infect different mosquito organs, beginning with sporadic infections in the midgut epithelium [52,53]. After infectious foci involving multiple cells are formed and subsequently merge throughout the entire midgut, the virus may disseminate to other organs and target salivary glands as a destination in which viral replication can also occur [54]. The complete route of viral dissemination in the mosquito may differ based on the virus strain [55]. Progeny virions that accumulate in salivary glands can thus be transmitted to a naive human host through the next bite for a blood meal [56]. As for the process of virus infection in the mosquito vector, various barriers must be overcome, including infection and escape barriers in the midgut and infection and escape barriers in the salivary glands [20]. Since huge numbers of progeny viruses are produced in the mosquito, it serves as a “virus manufactory”.

Effects of DENV infection on mosquito cells such as C6/36 cells relatively differ from those on mammalian cells. In general, cell death or apoptosis of mammalian cells is induced by DENV infection due to a prolonged shutdown of protein synthesis and persistent virus-induced oxidative stress within the cell [57,58]. Once progeny virions burst out from dead cells in the bloodstream or cell culture, they normally become a new source of infection for the next wave of spread to neighboring cells [59]. This reflects the ability of a virus to utilize the “release-and-entry” mode to spread the infection among mammalian cells. Despite this, the cell-to-cell transmission mode was observed for several human viruses, especially in patients with chronic infection [60,61]. Hepatitis C virus (HCV) is a good example, as it can establish a chronic infection in the liver, and cell-to-cell transmission was demonstrated to be an efficient way for viral spread in the liver [62,63]. The human immunodeficiency virus (HIV) generally infects a cluster of differentiated 4-positive (CD4^+^) memory T cells of the immune system by cell-free virions [64]; the cell-to-cell mode is usually utilized during intercellular transmission as it is more efficient and rapid [65]. Herpesviruses (HSVs) also rely on cell-to-cell transmission for spread to take advantage of evasion from the effect of neutralizing antibodies [66,67]. It is extremely important for the virus to escape adaptive immune responses generally activated through a process known as antigen presentation [68].

**Figure 1:**
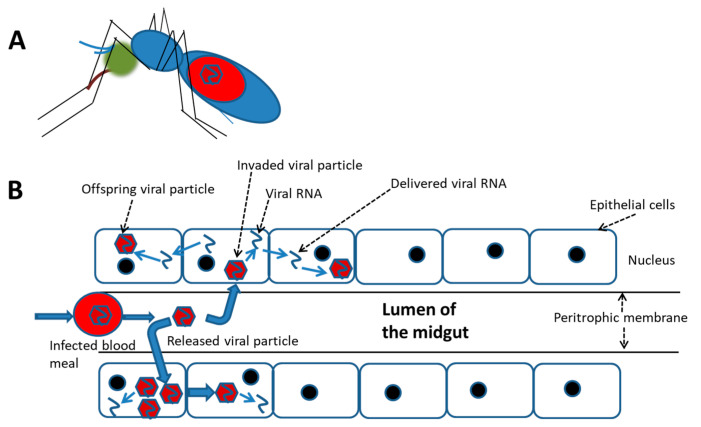
A schematic cell-to-cell spreading model of the dengue virus ingested in accompany with the blood meal in the mosquito midgut. (**A**) The virus infection in the female mosquito vector begins with a blood meal from viremia of a patient. (**B**) Simulation for cell-to-cell spread of the virus in the midgut of an engorged female. At the beginning, the ingested virus sporadically infects epithelial cells of the midgut. It is followed by extensively spread via cell-to-cell transmission before the virus disseminates to other organs.

In contrast, a relatively high rate of infected mosquito cells was demonstrated to survive with trivial damage, e.g., a cytopathic effect (CPE) and ultrastructural alterations, which were frequently observed in mammalian cells [69]. Moreover, cellular molecules may be induced by DENV infection in mosquito cells; many of these promote cell homeostasis that keeps infected cells alive. This indicates that the “release-and-entry” mode for viral spread might not be essential for viral dissemination in and/or between mosquito tissues; alternatively, the “cell-to-cell” mode could be a better way for the virus to spread more efficiently and rapidly (Figure 1). DENV infection is generally initiated by cell-free viruses in the mosquito midgut, likely followed by the cell-to-cell mode of dissemination to their destination, i.e., salivary glands. In cultures of mosquito cells with DENV2 infection, virions and viral RNA carried by vacuoles (VCs) were demonstrated to transfer from one cell to its neighbors via the cell-to-cell transmission mode [70,71]. During the transmission process of this virus, a novel tetraspanin called C189 is induced and is incorporated as a membrane component of VCs (C189-VCs) formed in response to DENV infection in mosquito cells [72]. The VC is presumed to serve as a vehicle which carries the virus from one cell to its neighbors. Moreover, a “viral synapse” (VS) located in the intercellular space is frequently formed [71], which was also observed at the interface between donor and recipient cells during cell-to-cell transmission of HIV [65]. The formation of VCs in humans may play a role of helping the virus evade humoral immunity, resulting in rapid transmission and continued infection of the host [73]. As a matter of fact, cell-to-cell transmission can enhance the efficiency of DENV dissemination within the mosquito because severe damage to cells is usually not evident during infection.

## 4. Innate Immunity and Its Signaling Pathway in the Mosquito

In order to maintain a continuous cycle of transmission of arboviruses in nature, survival with normal activities of the infected invertebrate host, such as flight and blood-feeding, remaining intact is essential. It is known that mosquitoes and other invertebrates have no recognized adaptive immunity as generally seen in vertebrates [74]. Alternatively, mosquitoes rely entirely on their innate immune system to withstand microbial infections caused by viruses, bacteria, fungi, and parasites [75]. Innate immunity in the mosquito is now known to initiate immediate protection against infections through humoral and/or cellular responses, which are usually induced by the invading microbe [76].

Conventional innate immune signaling pathways against pathogens in insects include the Toll, immune deficiency (IMD), and Janus kinase-signal transducer and activator of transcription (JAK-STAT) pathways [77]. The *Toll* gene was first discovered in the early stage of *Drosophila* embryos, where it functions as a regulator of dorsoventral axis formation [78]. It was found to have additional functions of regulating innate immune responses in larval and adult stages of *Drosophila* [79]. In fact, the Toll pathway is also induced by infections of fungi and Gram-positive bacteria [80]. Currently, at least 11 *Toll* genes and related proteins are known to be encoded by the *Drosophila* genome [81]. Concepts applied to the innate immunity of mosquitoes were mainly obtained from observations of the fruit fly *Drosophila melanogaster*, and functions of antiviral defense are thought to generally be conserved [22,82]. It was found that DENV infection can activate transcription of Toll pathway components in *Ae. aegypti* tissues, such as the midgut and salivary glands [83]. Ultimately, the Toll pathway plays a significant role in cellular resistance to infection, eventually leading to control of viral infections in the mosquito.

The IMD pathway is generally involved in the defense against Gram-negative bacteria [84]; it is activated through a cascade of events similar to those in the Toll pathway [83]. Both the Toll and IMD pathways are known to induce antimicrobial proteins (AMPs) and some so-called putative effectors [85]. AMPs are formed in fat bodies and are released into the hemolymph to kill invading microbes [86]. As is currently known, AMPs, including defensin, cecropin, and transferrin, are formed and act as antiparasitics as observed in the *Ae. aegypti* mosquito when infected with the nematode *Wuchereria bancrofti* [87]. The IMD pathway is also important in defense against infections by bacteria and *Plasmodium* parasites in mosquitoes [88]. The IMD pathway was recently reported to play a role in the antiviral response in mosquitoes, as upregulation of IMD components and effectors was identified when a mosquito was infected by the DENV or Sindbis virus (SINV) [22]. Recently, it was found that the IMD pathway is required for anti-DENV defense in refractory strains of *Ae. aegypti* [83]. The JAK-STAT pathway in insects was first characterized in *Drosophila* due to its role in development [22]. Further, the JAK-STAT pathway was linked to immunity against bacterial infections in *Anopheles gambiae* and *Drosophila* [89]. Activation of the JAK-STAT immune signaling pathway was also demonstrated to control DENV infection particularly in the midgut of *Ae. aegypti* [90]. This reflects the antiviral role of the JAK-STAT immune signaling pathway in the mosquito. Further, a panel of mosquito genes in relation to the JAK-STAT pathway was found to be triggered in *Ae. aegypti* by DENV infection. This reflects the possibility that an anti-dengue mechanism can be induced in the mosquito vector [24,90].

The RNA interference (RNAi) pathway is an additional mode of immunity that refers to RNA-mediated regulation of gene expressions; it was first discovered in the nematode *Caenorhabditis elegans* after injection of corresponding double-stranded (ds)RNA to silence specific genes [91]. It originally represented silencing of endogenous genes by introducing exogenous dsRNA with the same sequence as the gene to be silenced [92]. Curently, three gene-regulatory pathways are known to comprise the RNAi mechanism: Small RNAs, including small interfering (si)RNA, microRNA (miRNA), and Piwi-interacting (pi)RNA pathways are utilized to mediate RNAi. As a result, the RNAi pathway may consequently play roles in regulating arbovirus-vector interactions and controlling virus replication [22,92]. Arthropod vectors preferentially select innate responses to limit viral replication and avoid damage due to an infection. Each signaling pathway of innate immunity of insects strikes against invading microbes, and the various pathways may work as a network in order to produce a better effect against microbial invasion [93].

## 5. Endoplasmic Reticular (ER) Stress and the Unfolded Protein Response (UPR) Induced by DENV in Mosquito Cells

The ER is an organelle that serves as the site for multiple post-translational modifications, including disulfide bond formation, proper folding and glycosylation of proteins, specific proteolytic cleavages, and assembly of multimeric proteins [94]. Newly synthesized proteins need to remain here for proper arrangement of their conformations and resultant functions. However, ER stress can be induced and cause harmful damage to cells if proteins are misfolded or unfolded, as they accumulate in the ER lumen and fail to be eliminated to the cytosol for degradation [95,96,97]. Reactive oxygen species (ROS) are produced by living organisms and cells during the process of normal cellular metabolism [98,99]. Under a condition of ER stress, ROS accumulation damages cells [100]. Most ROS are free radicals that possess a single unpaired electron located in an outer orbit [101,102], and those include the superoxide anion (O_2_^•−^), hydrogen peroxide (H_2_O_2_), hydroxyl radical (^•^OH), and singlet oxygen (^1^O_2_) [100]. On the other hand, appropriate amounts of ROS, usually low to moderate concentrations, are essential for a number of cellular physiological processes such as cell growth, differentiation, apoptosis, and signaling activation [103]. Failure to keep ROS in a balanced status may induce oxidative stress within cells and result in adverse modifications to cell components, i.e., lipids, proteins, and DNA [104]. Cell death may occur upon accumulation of higher levels of oxidative stress within a cell [104,105]. In order to survive ER stress, the UPR is initiated to restore cell homeostasis [106]. ER stress can also be induced by invading DENVs in both mammalian and mosquito cells. However, cells from these two different organisms generally end up with different fates due to different effects of the UPR being activated after stimulation by ER stress in different infected cells.

Apoptosis usually occurs in cells derived from vertebrates when ER stress persists [107]. As mentioned above, mosquito cells usually survive ER stress induced by DENV infection because of induction of the UPR during infection [108]. It generally helps alleviate ER stress and prolong cell survival [109,110], indicating a positive effect of the UPR in DENV-infected mosquito cells. The UPR is a sequence of reactions working through a relatively sophisticated signaling system [111]. There are three branches of UPR signaling cascades that work on mitigating ER stress; these are mediated by transmembrane sensor proteins including protein kinase-like ER resident kinase (PERK), activating transcription factor 6 (ATF6), and inositol-requiring enzyme 1 (IRE1) [112]. These ER-transmembrane proteins are physiologically bound to the ER-resident binding immunoglobulin protein (BiP)/glucose-regulated protein 78 (GRP78). Dissociation of BiP/GRP78 from transmembrane proteins in response to the UPR is essential for triggering downstream regulatory activities under ER stress [113]. BiP/GRP78 reside in the ER and were found to increase its expression in mosquito (C6/36) cells infected with DENVs [114].

Both *GRP78/BiP* and *GRP94* (also known as endoplasmin) chaperones are commonly used as ER stress sensor genes, since they can be sensitively induced by ER stress, including that induced by viruses, e.g., a rotavirus in a rhesus monkey kidney cell line [115]. By examining BiP/GRP78 levels in C6/36 cells with DENV2 infection, a peak (5.20-fold) was reached at 24 hpi [114]. This suggests that DENV2 in mosquito cells, as in mammalian cells, activates the UPR to cope with ER stress in the early stage of infection. It is known that upregulation of BiP/GRP78 is mediated by the spliced form of the X-box-binding protein-1 (XBP1) transcription factor which is activated by ER to nucleus signaling 1 (IRE1) [72,116]. This process usually creates an intracellular status beneficial for cell survival and virus propagation in the early stage of virus infection in both insect and mammalian cells [117,118]. However, death of mammalian cells, but not mosquito cells, may occur when ER stress continues for a longer time. This indicates that mosquito cells can tolerate or alleviate continued ER stress induced by the virus, leading to a significantly higher increase in the survivability of infected cells.

## 6. First-Line Antioxidant Defense in Mosquito Cells with DENV Infection

Adult females of most mosquito species feed on the blood of vertebrate hosts which they prefer in order to meet their need for nutrients and egg production. The high temperature of the blood meal and digestion of ingested blood in the midgut can result in the release of pro-oxidant molecules that may be toxic to the mosquito [119,120]. In spite of this, engorged female mosquitoes are not damaged by the process of this essentially normal characteristic of their reproductive physiology. Antioxidant defense induced in the mosquito (cells) was reported to protect mosquito tissues from challenges due to oxidative stress [121]. This indicates that the mosquito has an innate ability for protection by alleviating oxidative stress induced by a blood meal.

Blood feeding by the mosquito vector also provides an opportunity for arboviruses to be transmitted between humans and/or animals. The accompanying virus ingested with the blood meal actually causes a stress cascade to form in the mosquito; it also eventually occurs in humans and other vertebrate hosts. Increased oxidative stress derived from the accumulation of ROS was implicated in viral pathogenesis [122]. It generally causes an alteration of the oxidation/reduction (redox) status in DENV-infected human cells [123]. The resultant disruption of cellular homeostasis leads to cell damage and even death if the antioxidant barrier is insufficient [124]. The viral inflammatory process was found to cause alterations or turbulence in the antioxidant system in patients with classic dengue fever, implying an association between antioxidant activity and the disease [125]. The relationship between oxidative/antioxidative responses and dengue pathogenesis was recently suggested. In particular, expression levels of endogenous antioxidant genes including catalase (CAT), manganese superoxide dismutase (MnSOD), and glutathione peroxidase (GPX) were found to be downregulated in dengue patients. Of these, significant downregulation of MnSOD expression was recorded in patients with secondary dengue infection [126]. More results from dengue patients also showed significantly lower levels of the plasma Trolox-equivalent antioxidant capacity, serum paraoxonase, and erythrocyte-reduced glutathione (GSH) and glutathione peroxidase (GPx) activities [127]. Moreover, lower SOD levels were found in humans with chronic HCV infection [128]. Collectively, dengue infection in humans usually results in an insufficiency of endogenous antioxidant enzymes which leads to the formation of oxidative stress and resultant disease. In fact, antioxidants have been used to treat chronic HCV infection [129].

**Figure 2:**
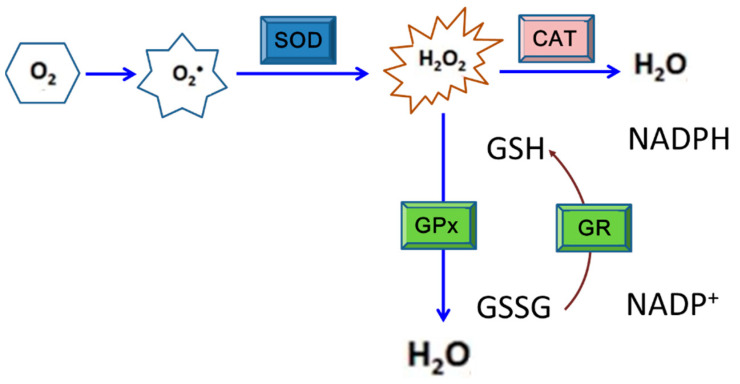
The enzymatic antioxidant system consisting of superoxide dismutase (SOD), catalase (CAT), glutathione peroxidase (GPx), and glutathione reductase (GR) that works for cell protection under oxidative stress. This is the principal defense system against reactive oxygen species (ROS) exposure in most organisms. SOD is able to catalyze the reaction to decompose superoxide anion radicals to H_2_O_2_, which is then converted to water and oxygen by CAT or GPx. Generally, CAT is the most efficient redox enzyme that catalyzes the conversion of H_2_O_2_ to water and oxygen. Alternatively, H_2_O_2_ can be removed via oxidizing glutathione to become GSSG by GPx; in which GR is also involved.

Like most mammalian cells, DENV-infected cells also produce ROS, principally in earlier stages of the infection. Nevertheless, the ROS-induced damaging effects do not occur in mosquito cells as observed in mammalian cells [130]. As a place for DENV replication and transmission to a human host, this phenomenon is an evolutionarily logical inference (Figure 2). As a result, elimination or removal of ROS from infected mosquito cells is required before the harmful effects derived by the accumulation of oxidative stress occur in those cells [98]. According to recent studies, antioxidants, including SOD, CAT, GPx, glutathione S-transferase (GST), glutaredoxin (Grx), thioredoxin (Trx), and protein disulfide isomerase (PDI), were detected to have higher gene expression levels in mosquito cells in response to DENV2 infection [108,114,121,131]. Among these antioxidants, SOD, CAT, and GPX are defined as the first line in the entire grid of antioxidant defense [132]. Results indicated that upregulation of antioxidants may play a critical role in avoiding ROS accumulation that frequently affects cell differentiation, cell growth, apoptosis, and defense against microbes [133]. Lack of sufficient antioxidant activities may cause apoptosis, particularly in mammalian cells with DENV or other arboviral infection.

SODs are metalloproteins that convert 2 superoxide anions into a molecule of hydrogen peroxide (H_2_O_2_) and one of oxygen (O_2_) [134]. According to an investigation of mosquito cells with DENV2 infection, SOD activity was detected to have significantly increased at 24 h post-inoculation (hpi), and higher levels persisted to 48 hpi. This indicates that SODs may be involved in stress alleviation and thus help mosquito cells evade harmful effects caused by DENV infection. On the other hand, SOD activity in BHK-21 cells also increased to as a high level as that shown in mosquito cells at 24 hpi. However, its activity had sharply fallen to about half that amount by 48 hpi, indicating that the antioxidant defense would not continue to protect mammalian cells during DENV infection [108]. A further study also showed that accumulation of ROS resulted from disruption of the balance between oxidative stress and the antioxidant response as usually shown in the latter stages of DENV infection (>24 hpi); by this time, redox homeostasis may have collapsed in response to the infection in mammalian cells [135]. H_2_O_2_ that usually forms due to oxidative stress is converted to H_2_O via the activity of CAT, a heme protein principally located in peroxisomes and in the inner mitochondrial membrane [136]. The conversion of H_2_O_2_ into H_2_O can also be implemented via activity of GPx, a family of cytosolic selenoenzymes with peroxidase activity; this also helps protect organisms and cells from oxidative damage [137]. In fact, the GPx expression level in DENV-infected mosquito cells was found to have increased to about 2.5- and 5-fold by 24 and 48 hpi, respectively, even higher than the level of SOD [108]. This suggests that GPx is also one of the responsive members of antioxidants in mosquito cells infected by DENV.

## 7. The Effect of GST in Mosquito Cells

GSTs consist of a group of multifunctional proteins that are widely distributed in eukaryotes and prokaryotes [138]. These enzymes utilize glutathione to metabolize hydrophobic toxic substance such as insecticides, drugs, and toxic endogenous substrates, and are generally active in phase II of toxic metabolism [139]. GSTs specifically catalyze the conjugation of GSH to electrophilic centers of exogenous and endogenous xenobiotics, rendering the compounds more water-soluble. This kind of cellular detoxification against xenobiotic compounds is also implemented by many cells that are able to survive oxidative stress [140]. Cancer cells frequently use this process to acquire drug resistance [141].

GSTs in eukaryotes can be divided into at least three major groups of proteins according to their cellular localization, i.e., cytosolic, mitochondrial, and microsomal groups [140]. Cytosolic GSTs include most members of this superfamily, and they usually project their relevance on human diseases. These can be further classified into subclasses including alpha, zeta, theta, mu, pi, sigma, and omega on the basis of primary amino acid sequence similarities and substrate specificities [140]. Cytosolic GSTs found in insects belong to the six major subclasses of delta, epsilon, omega, sigma, theta, and zeta, and there are still several unclassified genes [142,143]. Among these, the delta and epsilon groups are insect-specific and have evolved to serve in detoxification, particularly that associated with insecticide resistance [144]. Thus far, GSTs have been identified from more than 24 species of insect [145]. At least 26 of ca. 30 GST members found in mosquitoes are present in *Ae. aegypti* which is the major vector of dengue fever and several other arboviruses [146,147]. Despite differences in classification, mosquito GSTs are suggested to have common pathways involved in detoxification that result in resistance against insecticides. It turns out that both gene expressions and enzymatic activities of GSTs are induced at higher levels in resistant strains of insects [148,149].

Insecticides entering the body of insects can destroy the redox balance and thus cause oxidative stress reactions. However, they can be eliminated by some of the GSTs [150,151]. This implies that GSTs can protect insect cells against ROS-induced cell death [145]. Among etofenprox-selected individuals of *Nilaparvata lugens* with rice ragged stunt virus (RRSV) infection [152], activities of GSTs usually increased when the infection rate of RRSV was low [153]. This suggests that GSTs are important in producing an environment beneficial for viral defense. In silkworms infected with *Bombyx mori* nuclear polyhedrosis virus (BmNPV), a significant decrease in GST was shown in fat bodies and the midgut of a BmNPV-susceptible strain. In contrast, GST activity increased at 24 and 48 h in the midgut and at 72 h in fat bodies of a BmNPV-tolerant strain [154]. This reveals that higher GST activity is closely related to the inhibition of BmNPV infection in the silkworm. It seems that GSTs can function to protect insects from xenobiotics and invasive microbes.

In a similar manner, significant induction of GST was demonstrated in mosquito cells infected with DENV2 [108]. Moreover, changes in the mitochondrial membrane potential (MMP), a signal of apoptosis, were seen in mosquito (C6/36) cells at 24 hpi, indicating that ER stress may been induced as that usually shown in DENV-infected mammalian (BHK-21) cells [108]. Importantly, higher levels of gene expression and enzymatic activity of GST were seen in C6/36 cells but not in BHK-21 cells. By 24 hpi, the GST activity in BHK-21 cells had rapidly decreased. This suggests that DENV-induced GST activity in mosquito cells may create an environment with less oxidative stress, which would be beneficial for viral amplification. The *GST* gene in C6/36 cells was identified, and it was evidently upregulated in response to DENV2 infection via analysis of DNA sequences derived from subtracted clones selected by dot blot array screening [72]. When GST was knocked-down in DENV-infected C6/36 cells, the concentration of SOD, an antioxidant gene, was elevated 1.52-fold compared to that in mock-infected cells [108]. This suggests that GST plays a critical role in relieving oxidative stress and thus protects C6/36 cells from viral infection. Furthermore, most infected C6/36 cells were arrested at the sub-G_1_ phase (M_1_) of the cell cycle at 36 and 48 hpi as measured by propidium iodide (PI) staining [155]. Apparently, GST is an effective factor involved in reducing cell death caused by DENV2-induced oxidative stress in mosquito cells [156,157]. Despite that GST has been involved in catalyzing the conjugation of GSH on a wide variety of substrates as mentioned above, the role of GST as a protective effector in the case of viral infections remains to be clarified thus far. Perhaps it participates in detoxifying endogenous compounds such as unfolded or incorrectly-folded proteins, resulting in alleviation of virus-induced oxidative stress.

## 8. The Role of Eukaryotic Translation Initiation Factor 5A (eIF5a) in Protecting Mosquito Cells from DENV Infection

eIF5A, formerly called eIF-4D, is an acidic protein relatively conserved across species from yeast to humans [158]. It is the only protein currently known to contain the amino acid, hypusine (*N*ε-(4-amino-2-hydroxybutyl) lysine), which is an unusual amino acid derived from a modification of lysine by spermidine [159]. The eIF5A protein was originally considered to be a translation initiation factor [160]. However, its role in translation is controversial since its deletion in yeast results in an insignificant decrease in the synthesis of total proteins [161]. Despite this, eIF5A was found to possess functions in relation to cell proliferation, cell viability, and cell-cycle progression [160]. It was also reported to have an additional function of serving as a nucleocytoplasmic shuttle for some messenger (m)RNAs associated with cell division [162]. A remarkable decrease in eIF5A abundance was seen during aging of the adult *Ae. aegypti* [163]. Free radicals induced during the progression of aging were reported to regulate eIF5A transcription [164]. In C6/36 cells with DENV infection, *eIF5A* was a gene selected through the method of polymerase chain reaction (PCR)-select complementary (c)DNA subtraction, and its upregulation of mRNA and protein expressions was validated [72]. eIF5A expression was not significantly induced by ultraviolet (UV)-inactivated DENV2 compared to that detected in cells infected with the untreated virus, i.e., without UV inactivation [165]. This suggests that eIF5A activity can only be induced by a virus replicating in the cell.

Based on an analysis of the cell cycle, C6/36 cells, either infected with DENV2 or not, tended to remain in the S phase, which is a phase suitable for mosquito cell replication, protein synthesis, and even maturation of the virus [165]. A previous study that reduced eIF5A expression showed induction of G_1_ arrest in mammalian HeLa cells [166]. This suggests that eIF5A in virus-infected mammalian cells also induces G_1_ arrest, thereby increasing the cell death rate. When DENV2-infected C6/36 cells were treated with ciclopirox olamine (CPO; a functional inhibitor of eIF5A), a slightly increased cell death rate of 5.85% was recorded at 24 hpi. However, a rapid increase was reported at 48 hpi (up to 28.04%), which significantly differed from that observed in cells infected with DENV2 but not treated with CPO [165]. This result revealed that the survival of mosquito cells during DENV2 infection may be due to progression of the normal cell cycle. Actually, eIF5A might be involved in this process because of its function as a regulator of the enhanced expression of p53 in response to viral infection [107]. ROS generated by cells as products or byproducts usually act as upstream signals that trigger p53 activation and/or downstream factors that mediate cellular proliferation or apoptosis [167,168].

p53 activated by low-intensity oxidative stress normally triggers an antioxidant response, while p53 may activate pro-oxidant targets during high-intensity intracellular stress [169]. As a result, p53 can be a gene with dual roles via conversion from a killer into a healer based on the intensity of stress-induced ROS [170]. Exposure to cellular stress can trigger p53 to induce cell growth arrest or apoptosis in mammalian cells, and thus it can serve as a tumor suppressor [169]. p53 identified in mammals is structurally unique, as it lacks the sterile alpha motif (SAM) domain which exclusively exists in the C-terminal region of two other homologues, i.e., p73 and p63 [171,172,173]. The SAM is a putative protein–protein interaction domain and exclusively exists in the C-terminal region of p73 and p63 in mammals; it is required to stabilize the structure of the carboxyl-terminal oligomerization domain (OD) [172]. The OD contains a nuclear export signal (NES) and contributes to the formation of a dimer out of two dimers of p53 in structure [174]. Evolutionally, p53, p63, and p73 are members of the p53 family, although they possess different physiological functions [175]. p53 has been identified in a variety of insects and other invertebrates, such as *Drosophila melanogaster* and *Caenorhabditis elegans* [176]. *Drosophila* p53 is known to be functionally similar to that identified in mammals; both trigger apoptosis under stressful conditions [177]. More evidence revealed that invertebrate p53 can also function in DNA repair, cell-cycle checkpoint responses, and even cell differentiation; these reflect that p53 could be beneficial for invertebrate cells to survive specific conditions in the cell [178].

p53 was recently identified from mosquitoes such as *Culex quinquefasciatus*, *Ae. aegypti*, *Ae. albopictus*, and *Anopheles* species [173]. Similar to that from mammals, the structure of p53 identified from the mosquito lacks the SAM domain. Very importantly, p53 identified from C6/36 cells was significantly upregulated in response to DENV2 infection. When the *p53* gene is knocked-down in DENV2-infected C6/36 cells with a fragment of synthesized dsRNA, both superoxide anions and hydrogen peroxide had significantly increased at 48 hpi [51]. This suggests potential relevance of mosquito p53 which may function in cell protection. Although most invertebrates possess a single homolog of p53 family genes [179,180,181,182], two isoforms, i.e., A and B, of p53 are found in *D. melanogaster*. However, only isoform A was confirmed to be involved in mediating the apoptotic response [183]. This indicates that there must be a functional difference between the two isoforms. Two p53 paralogues, i.e., p53-1 and p53-2, were identified in C6/36 cells on the basis of a phylogenetic analysis [173]. Functional assays revealed a relevant difference in their response to the DENV infection. Furthermore, the expression of p53-2 but not p53-1 was significantly upregulated in C6/36 cells in response to DENV2 infection [173]. There were obvious cell death rate increases in C6/36 cells with p53-2-knockdown and DENV2 infection at 48 hpi. In the meantime, evident changes in ROS, including superoxide anions and hydrogen peroxide, were induced in C6/36 cells with the same treatment especially at 48 hpi [51]. In further observations of responses of selected genes associated with antioxidant defense, CAT obviously decreased in C6/36 cells with infection by DENV2 and p53-2-knockdown [51]. Looking at the entire process of cell protection from DENV infection, virus-induced eIF5A may act as an initiator that upregulates p53-2 and subsequently CAT. CAT is known to metabolize hydrogen peroxide and also reactive nitrogen species [184]. This reveals that ROS-regulated p53 and its downstream genes could trigger an antioxidant cascade that is responsible for protecting mosquito cells from DENV infection. As a matter of fact, the entire process might be initiated by upregulation of eIF5A due to viral infection.

## 9. Avoidance of Cell Death in DENV-Infected Mosquito Cells

Arboviruses require mosquitoes or other arthropods as vectors for effective transmission between mammals. This shows the importance of mosquito survival from stress induced by blood-feeding, and certainly also by infections with viruses and other microorganisms that accompany the blood meal. In order to survive during viral infection, mosquito cells must have co-evolved with viruses to resist the deleterious effects of the oxidative stress induced by viral infection. As mentioned above, induced oxidative stress can cause changes in the MMP due to a transition in mitochondrial permeability [185]. It was repeatedly demonstrated that DENV2 infection can induce a significant increase of the MMP in both BHK-21 and C6/36 cells at 24 hpi compared to that in mock-infected cells [108]. In addition, DENV infection also causes dysregulation of Ca^2+^ in the cytosol and mitochondria, leading to alterations of cytosolic calcium ions ([Ca^2+^]cyt) in infected cells [186]. The influx of Ca^2+^ through the inner membrane is regulated by a highly selective Ca^2+^ channel known as the mitochondrial Ca^2+^ uniporter that modulates Ca^2+^ uptake based on the MMP [187]. Cytochrome C oxidase is an enzyme in the mitochondrial electron transport chain that works by driving oxidative phosphorylation [188]. It seems to play a role in stress-induced apoptosis in insects with viral infection [189]. Opening of the mitochondrial permeability transition pores induces depolarization of the transmembrane potential, leading to the release of apoptogenic factors [190]. Taken together, mitochondrial dysfunction is a sign and eventually plays a central role in the apoptotic pathway [190]. In fact, changes in [Ca^2+^]cyt were detected in C6/36 cells with DENV2 infection as early as 24 hpi, implying that an early signal of apoptosis is shown in DENV2-infected mosquito cells as also seen in mammalian cells [114]. It was mentioned that C6/36 cells with DENV2 infection frequently remain in the S phase of the cell-cycle, further implying there might be an antiapoptotic effect which plays a crucial role in protecting mosquito cells during infection.

To prove this possibility, *inhibitor of apoptosis* (*IAP*) gene expression was measured and showed an increase of 3.8-fold at 48 hpi in DENV2-infected BHK-21 cells; on the contrary, it decreased to 60% at the same time point of infection [131]. It is known that both mosquito and human cells are susceptible to DENV2 infection. However, most infected mammalian cells end up dying, suggesting that the IAP might be a critical factor deciding the fate of cells infected with the virus. The IAP is structurally composed of two baculoviral IAP repeat (BIR) domains and a RING-finger domain at its carboxyl terminus [191]. It is a group of proteins that mainly function by blocking the intrinsic apoptosis pathway, leading to cell survival [192]. Within cells, the role of the IAP is to bind and thus block caspases, a family of cysteine proteases that play integral roles in the apoptotic process [193]. Measuring activities of caspases in both mammalian and mosquito cells with DENV2 infection revealed no evident activation of caspase-9 (the initial caspase) or caspase-3 (the effector caspase) in the former (C6/36 cells) at 48 hpi. In contrast, respective 3.3- and 3.4-fold increases in caspase-9 and -3 were shown in BHK-21 cells [131]. Caspases are sequentially activated to form an apoptosome that leads to the occurrence of apoptosis [194]. Furthermore, activities of both caspase-9 and -3 were significantly elevated in C6/36 cells with IAP-knockdown at 48 hpi; this led to an 8.3% increase in the apoptosis rate [131]. This further suggests the critical importance of IAP in avoiding apoptosis in mosquito cells with viral infection. This shows that apoptosis induced by DENV2 infection is dependent on the mitochondrial pathway that begins with a change in the permeability of the mitochondrial outer membrane [195]. As a matter of fact, it occurs only in mammalian and not in mosquito cells in response to DENV2 infection. When C6/36 cells were treated with double-knockdown of IAP and GST, respective increases of up to 2.6- and 2.83-fold in caspase-9 and -3 during infection for 48 hpi were detected. This resulted in a significant increase in the apoptosis rate of up to 23.52%, which was significantly higher than that of cells with GST-knockdown only [131]. In the meantime, IAP was found to be positively regulated when the antioxidant status improved in mosquito cells by adding antioxidant reagents [131]. Presumably, the apoptosis rate is alleviated through upregulation of IAP that binds to caspases-9 and -3. In turn, IAP is important due to its role as a regulator to inhibit activation of caspases and thus downregulate cell death pathways [196]. Very likely, this process provides a critical step for mosquito cells that allows them to resist cell death from DENV2 infection [197].

## 10. Conclusions

Viruses, mosquitoes, and humans are three entities with different potentials for evolutionary change. A complex contributing to viral replication by adjusting evolutionary interactions among them is formed to complete the transmission cycle of many arboviruses in nature [14]. Viruses have developed a sophisticated but effective strategy that works to hijack cellular factors for their sustained maintenance in nature during the long pathway of coevolution [198]. It is particularly important for the mosquito to be able to bear the burden of oxidative stress induced by viral infection to protect cellular growth and produce viral progeny [199]. Theoretically, the mosquito exhibiting normal physiological behaviors is based on the capacity of each infected cell to adapt itself to the virus-induced oxidative stress.

**Figure 3:**
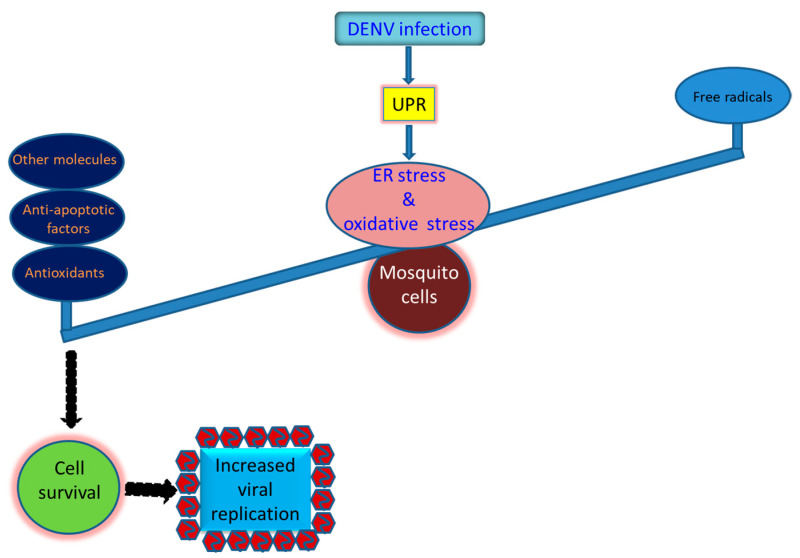
Strategies that utilized by the mosquito cells to survive the infection by the dengue virus and other arboviruses. Prosperous replication of the virus in mosquito cells is required for its sustainability in nature. However, it is dependent on the mosquito vector that can bear the burden of the oxidative stress induced by the virus infection, resulting in normally cellular growth and productive virus replication. Antioxidant defense in infected mosquito cells is generally induced to adjust the redox status of the cells to adjust cellular homeostasis. Antioxidants including SOD, CAT, and GPX are commonly upregulated in mosquito cells infected by the dengue virus. The anti-apoptotic effect involving upregulation of the inhibitor of apoptosis (IAP) and resultant reduction of caspase-9 and -3 activity can also be critical for infected cells to survive. More, other molecules such as glutathione S-transferase (GST), eukaryotic translation initiation factor 5A (eIF5a), and one paralogue of p53 (p53-2) can also be beneficial for mosquito cells to resist oxidative stress induced by the viral infection.

A number of arboviruses require mosquitoes as vectors to transmit them between vertebrate hosts. Therefore, mosquito cells are required to survive and produce a higher level of viral load during infection. Antioxidant defense is generally induced to adjust the redox status of cells under stress, resulting in cellular homeostasis that usually decreases virus-induced oxidative stress in mosquito cells. A variety of antioxidants that are upregulated during DENV2 infection have been identified in mosquito cells (Figure 3). Of these, SOD, CAT, and GPX are defined as the first line of the defense grid that copes with oxidative stress induced in mosquito cells infected with DENV. Other relevant molecules, including GST, eIF5A, and p53-2, identified in DENV2-infected mosquito cells are also involved in stress reduction and protection of infected cells. An antiapoptotic effect is also important to avoid the death of mosquito cells during infection. The occurrence of this phenomenon is generally initiated by upregulation of IAP that binds caspases-9 and -3, resulting in a reduction of apoptosis in mosquito cells. Taken together, survival from DENV infection is a requirement of mosquito cells and mosquitoes in order to support productive infection and sustain the viral transmission cycle in nature. Perhaps multiple strategies are needed, working independently or through an exquisite network, rendering the mosquito to be an effective vector of the virus it transmits in nature.
